# The mediating role of self-esteem in the association between locus of control and fear of failure and hope for success among women in five Arab countries

**DOI:** 10.3389/fpsyg.2026.1890383

**Published:** 2026-10-21

**Authors:** Elie Gedeon, Celine El Zoghbi, Leen Awarkeh, Christian Salameh, Amanda Geagea, Diana Malaeb, Amthal Alhuwailah, Mai Helmy, Tabassum Rashid, Muna Barakat, Feten Fekih-Romdhane, Souheil Hallit, Sahar Obeid, Marie Anne El Khoury

**Affiliations:** 1 School of Medicine and Medical Sciences, Holy Spirit University of Kaslik, Jounieh, Lebanon; 2 Department of Psychology and Education, School of Arts and Sciences, Lebanese American University, Beirut, Lebanon; 3 College of Pharmacy, Gulf Medical University, Ajman, United Arab Emirates; 4 Department of Psychology, Kuwait University, Kuwait, Kuwait; 5 Department of Psychology, College of Education, Sultan Qaboos University, Muscat, Oman; 6 Department of Psychology, Faculty of Arts, Menoufia University, Shebin El-Kom, Egypt; 7 School of Social Sciences, Arts and Humanities, Al Akhawayn University, Ifrane, Morocco; 8 Department of Clinical Pharmacy and Therapeutics, Faculty of Pharmacy, Applied Science Private University, Amman, Jordan; 9 The Tunisian Center of Early Intervention in Psychosis, Department of Psychiatry “Ibn Omrane”, Razi Hospital, Manouba, Tunisia; 10 Faculty of Medicine of Tunis, Tunis El Manar University, Tunis, Tunisia; 11 Department of Psychology, College of Humanities, Effat University, Jeddah, Saudi Arabia; 12 Department of Psychology and Education, School of Arts and Sciences, Lebanese American University, Jbeil, Lebanon

**Keywords:** achievement motives, Arab women, cultural psychology, locus of control, self-esteem

## Abstract

**Background:**

Achievement motivation is commonly described through two related but distinct tendencies: hope for success and fear of failure. Locus of control may be associated with these achievement motives through self-esteem, but these relationships have been examined mainly in Western and individualist contexts. This study examined whether self-esteem statistically mediates the association between locus of control and achievement motives among Arab women, within the broader context of collectivist values, relational self-construal and gendered family socialization.

**Methods:**

A multinational cross-sectional online survey was conducted between July 2024 and March 2025 among native Arabic-speaking women aged 18 years or older residing in Lebanon, Egypt, Oman, Palestine and Morocco. Participants were recruited using convenience and snowball sampling. Locus of control was measured using the Internal-External Locus of Control Short Scale, self-esteem using the Single-Item Self-Esteem Scale and achievement motives using the Revised Achievement Motives Scale. Mediation analyses examined indirect associations through self-esteem. Bivariate analyses were used to examine perceived parental son preference in relation to achievement motives.

**Results:**

A total of 1,963 women completed the questionnaire (mean age = 27.42 years). Self-esteem statistically mediated the association between internal locus of control and hope for success (indirect effect *B* = 0.22, 95% bootstrap CI [0.17 to 0.27]) and the association between internal locus of control and fear of failure (B = −0.06, 95% bootstrap CI [−0.10, −0.02]). For external locus of control, self-esteem statistically mediated its association with hope for success (*B* = −0.08, 95% bootstrap CI [−0.13 to −0.03]), while no indirect association was observed for fear of failure (*B* = 0.001, 95% bootstrap CI [−0.01 to 0.01]).

**Conclusion:**

In this cross-sectional convenience sample of Arab women, self-esteem statistically accounted for part of the associations between locus of control and achievement motives, particularly hope for success. The bivariate associations involving perceived parental male preference suggest that family gender socialization may be relevant to women’s achievement motivation, but these findings should be interpreted cautiously and separately from the mediation models. Because of the cross-sectional design, convenience and snowball sampling, and low internal consistency of the brief locus-of-control subscales, the findings should be understood as preliminary associations rather than causal evidence. Further studies using longitudinal and stronger measures are needed.

## Introduction

Understanding how individuals’ approach and respond to achievement-related situations is a central concern in motivational psychology. Yet the psychological processes underlying achievement motivation do not unfold in a cultural vacuum (Markus and Kitayama, 1991). Cultural values, gendered social structures, and collective norms may be relevant to the meaning individuals attach to control, competence, and accomplishment in ways that established Western-driven models may not fully capture (Henrich et al., 2010). Women in particular represent a theoretically significant group for this inquiry, given that the intersection of cultural expectations, gendered socialization, and achievement-related beliefs is especially pronounced in Arab societies, where women navigate competing pressures between expanding educational and professional opportunities on one hand and deeply embedded gendered role expectations on the other (Douki et al., 2007). Although the countries included in this study differ in their sociopolitical conditions, economic environments, educational opportunities, and gender norms, they also share broad cultural characteristics, including the centrality of family relationships, collectivist orientations and gendered socialization, that provide a meaningful regional context for examining these psychological processes. This study examines the association between locus of control, self-esteem, and achievement motives among women in five Arab countries, with the aim of testing whether self-esteem statistically accounts for the indirect association between control beliefs and motivational orientations in a cultural context characterized by collectivist values and distinct gendered expectations around achievement (Abu-Hilal et al., 2016).

### Achievement motivation

Achievement motivation involves the determination to set goals and work toward fulfilling them, it is shaped by enduring motivational tendencies (Feng and Yang, 2024). As per Atkinson’s achievement motivation theory, individuals grow two main motivational tendencies: striving toward success and avoidance of failure (Feng and Yang, 2024). These tendencies are referred to as approach motivation and avoidance motivation, respectively (Feng and Yang, 2024). The first reflects hope for success whereas the second is characterized by fear of failure in achievement-related situations (Feng and Yang, 2024). Research suggests that motivation fosters greater confidence in pursuing success and enhances individuals’ willingness to confront and persist through increasing difficulties (Feng and Yang, 2024).

Longitudinal studies also show that these achievement-related motivational orientations can have mental health consequences (Steare et al., 2024). In adolescents, stronger approach-focused goals may predict lower depressive symptom severity over time (Steare et al., 2024). On the other hand, avoidance-focused goals may predict higher depressive symptoms (Steare et al., 2024). However, achievement motivation is not always protective for well-being, particularly under sustained pressure (Schoch et al., 2018). A strong motive under chronic strain may intensify stress related outcomes (Schoch et al., 2018). It has been reported to exacerbate the relationship between work discontent and vital exhaustion, a marker of prolonged psychological strain (Schoch et al., 2018). However, the degree to which these motivational tendencies are expressed and associated with other psychological variables may vary across cultural contexts in which individuals are embedded.

### Achievement motives and locus of control

Prior research suggests that achievement motives and locus of control are practically and conceptually associated, for the reason that achievement driven behavior has been associated with beliefs that one’s own actions are related to one’s outcomes (Wolk, 1973). In other words, a more internal control orientation, has been associated with achievement-related motivational patterns (Wolk, 1973).

Locus of control can be defined as a person’s belief about the extent to which they can control events affecting their lives (Ribolsi et al., 2022). This concept was first described by Rotter as the perception of the relationship between one’s actions and the outcomes that they achieve (Ribolsi et al., 2022). This locus of control is widely considered a continuum with two opposing orientations: internal and external (Ribolsi et al., 2022). Internal locus of control refers to the belief that life events are largely contingent on one’s own efforts and behaviors, whereas external locus of control refers to the belief that life events are largely attributable to factors outside one’s control, such as fate, luck, or other external circumstances (Ribolsi et al., 2022).

Subsequently, believing that one’s outcomes are connected to one’s own actions may be associated with a stronger sense of control in achievement contexts which may be relevant to the observed association between internal locus of control and more adaptive achievement-related behavior (Carden et al., 2004). Consequently, locus of control has been associated with achievement-related outcomes and may be relevant to how individuals respond to and engage with academic challenges (Carden et al., 2004). For instance, evidence shows that locus of control is significantly related to academic achievement with higher academic scores reported among individuals with more internal locus of control compared to those who hold a more external orientation (Suraj et al., 2024).

### Locus of control, self-esteem and achievement motives

Because control beliefs have been associated with self-evaluation, a more internal locus of control has been associated with higher self-esteem. Accordingly, self-esteem may statistically mediate the association between locus of control and achievement related outcomes and motives (Aikpitanyi et al., 2022).

Self-esteem refers to the overall sense of self-worth and is often described as a global self-evaluation (Hein and Hagger, 2007). In research, this global form of self-esteem is most frequently assessed by the Rosenberg scale, a widely used self-report measure of self-worth (Hein and Hagger, 2007). It is important in achievement contexts as it is linked to an individual’s persistence, choices and success across a range of behaviors (Hein and Hagger, 2007). In addition, self-esteem is closely related to one’s perception of their own competence (Hein and Hagger, 2007). Therefore, low self-esteem may be associated with interpreting failure as a reflection of personal inadequacy and with greater avoidance of evaluative or achievement related situations (Zhang et al., 2018).

Avoidance reactions may also be related to wider control beliefs (AbuSabha and Achterberg, 1997). When individuals believe that their outcomes are mainly connected to their own actions versus external forces, this may be associated with how they assess setbacks and threats to self-esteem (AbuSabha and Achterberg, 1997). Since locus of control reflects beliefs about personal control over outcomes, it may also be linked to how individuals perceive their own abilities and self-worth (AbuSabha and Achterberg, 1997).

This provides a theoretical rationale for examining self-esteem as a potential statistical mediator of the association between locus of control and achievement motives (Aikpitanyi et al., 2022). Control beliefs are closely linked to self-evaluation, and research shows that a more internal control orientation aligns with higher self-esteem whereas a more external orientation is associated with lower self-esteem (Aikpitanyi et al., 2022). Self-esteem is relevant because it has been linked to persistence, achievement-related choices and success across a range of behaviors in achievement contexts (Hein and Hagger, 2007). Lower self-esteem may be associated with interpreting failure as personal inadequacy and with greater avoidance of situations in which ability is evaluated, potentially corresponding to more avoidance-focused responses in competitive situations (Zhang et al., 2018). Evaluating these relationships within specific sociocultural contexts may help to show how locus of control and self-esteem relate to achievement motives.

### Cultural context: gender, collectivism, and self-evaluation in Arab societies

Achievement motivation, locus of control, and self-esteem have been extensively studied in Western, individualist contexts, yet these constructs may carry different meanings and show different patterns of association in collectivist cultural settings. Hofstede’s cultural dimensions framework (Hofstede et al., 2010) and Triandi’s elaboration of individualism–collectivism (Triandis, 1995) highlight that in collectivist societies, the self is fundamentally defined in relation to the group, family, community, and social roles take priority over individual autonomy and personal accomplishment (Markus and Kitayama, 1991). These theoretical perspectives suggest that psychological constructs such as locus of control and self-esteem may be experienced and expressed differently across cultural contexts. In highly collectivist contexts, attributing outcomes to external forces, like family expectations, social circumstances or fate, may not reflect a deficit in perceived agency in the same way it does in individualist frameworks; rather, it may represent a culturally normative adaptive orientation toward interdependence (Hornsey et al., 2019; Triandis, 1995).

Although Arab countries are often described as sharing broad cultural characteristics, including relatively collectivist social orientations, strong family structures and the importance of interpersonal obligations (Dwairy, 2006), they are far from culturally homogeneous. Lebanon, Egypt, Oman, Palestine, and Morocco differ substantially in their political systems, economic conditions, educational opportunities, and gender norms. Accordingly, the present study does not assume that these societies are identical, but rather that they share broad regional cultural characteristics to justify examining psychological processes within a broader Arab context while acknowledging important within-region diversity. Within this broader context, previous literature suggests that factors like religiosity and family expectations may be relevant to how beliefs about personal control are understood and expressed in some Arab populations (Dwairy et al., 2006; Ghuloum and Al-Amin, 2024; Zakiy et al., 2024).

Gender role theory further complicates this picture in the Arab context. Arab societies remain among the most gender-stratified in the world. According to the World Economic Forum’s Global Gender Gap report (2024) (World Economic Forum, 2024), the MENA region ranks last out of all world regions in gender parity, with a score of 61.7%, and political empowerment is projected to take 169 years to reach parity at current rates of change. Importantly, this persists alongside rapidly expanding educational attainment for women, creating a distinctive structural tension between rising aspirations and enduring gendered constraints that is particularly relevant for understanding achievement motivation in this context (Greenberg and Sagiv-Reiss, 2013). Women continue to operate within social structures in which family obligations and gendered expectations often take precedence over individual accomplishment, and for many women education and work remain contested rather than established rights (Abu-Hilal et al., 2016; Douki et al., 2007; Dwairy et al., 2006). This tension may be relevant to differences in motivational orientations that are not fully captured by general models of achievement motivation. Accordingly, hope for success may reflect not only an individual motivational tendency but also aspirations that may be associated with broader social and family expectations. Although these contextual factors were not directly measured, they provide one possible framework for interpreting achievement motivation among Arab women.

Self-esteem, too, may be differently anchored in Arab cultural contexts. Research suggests that in collectivist settings, self-esteem can be relationally constructed, dependent on social belonging, family approval, and adherence to valued roles (Du et al., 2017; Dwairy et al., 2006). This means that the link between internal locus of control and self-esteem, well-established in individualist samples, may be modified in Arab contexts where a sense of self-worth is partly maintained through relational and communal sources even when personal control is perceived as limited. Taken together, these theoretical perspectives suggest that the association between locus of control, self-esteem and achievement motivation may not operate identically across cultural contexts. Because the present study did not directly measure cultural mechanisms or moderators, it examines whether associations reported primarily in Western samples are also observed among women in Arab countries.

One culturally relevant contextual factor discussed in the literature is parental gender preference, a documented phenomenon in Arab societies whereby parents express a cultural preference for male offspring (Douki et al., 2007; Guilmoto, 2015; Schady et al., 2008; Sharaf and Mansour, 2019). For girls who grow up aware of this preference, the psychological consequences may be twofold. Some may internalize the implicit message of lesser value, which may be associated with lower self-esteem and a more avoidance-oriented motivational profile (Siah, 2015). Others may report stronger achievement striving and approach-oriented motivation, potentially as part of efforts to affirm their self-worth. These theoretical perspectives suggest that parental gender attitudes do not uniformly depress girls’ self-evaluation or motivation but may be associated with different motivational and self-evaluative patterns depending on how individuals relate to that cultural message. Accordingly, perceived parental son preference was included as a culturally relevant contextual covariate.

### The present study

Research from the Arab region suggests that self-esteem among women is associated with contextual and psychosocial factors, supporting the relevance of examining these constructs in the region. For instance, previous work in Lebanon examining correlates of self-esteem in a community sample found that better emotion-related functioning and personal accomplishment were associated with higher self-esteem whereas indicators of psychological distress (depression, anxiety-related factors, burnout-related depersonalization and suicidal ideation) were associated with lower self-esteem (Obeid et al., 2019). In Morocco, research emphasizing women reported that self-esteem varies across age categories and may be associated with many factors such as education and physical health (Rhaouti et al., 2022). This study highlighted that self-worth is not static and may reflect life circumstances (Rhaouti et al., 2022).

Concurrently to self-esteem, achievement-related outcomes in Arab countries show strong gendered patterns that make women a relevant group to study. For instance, large-scale data from Saudi Arabia reported higher academic achievement among female students compared with male students in mathematics and science (Barry, 2019). This may indicate that achievement-related outcomes can be prominent among females in at least some Arab regions (Barry, 2019). At the same time, another study about locus of control in Lebanon has discussed how gendered social structures may be associated with beliefs about agency and control, which is relevant when considering the associations between control beliefs, motivational patterns and self-evaluation (Nasser and Abouchedid, 2002).

In this study, we focus on a female population drawn from five Arab countries including Lebanon, Egypt, Oman, Palestine and Morocco. This investigation among females is particularly relevant as motivational processes and self-evaluations may be framed by gender-specific social experiences and expectations (Abu-Hilal et al., 2016; Borham et al., 2023). Furthermore, examining these variables within an Arab cultural context contributes to the existing literature by extending the results beyond Western countries, where most research about this subject has been conducted (Greenberg and Sagiv-Reiss, 2013). The multinational sample allows a wider comprehension of these constructs across different sociocultural settings within the same region.

Despite the fact that previous research separately linked achievement motivation, locus of control and self-esteem, less attention has been given to how these variables are associated with one another. Control beliefs may be associated with an individual’s self-evaluation, which is also associated with motivation to approach success or avoid failure (Baskaya et al., 2023; Hosseini et al., 2016). From this point of view, self-esteem may statistically account for part of the association between locus of control and achievement motives. With that being the case, our study aims to evaluate whether self-esteem statistically mediates the associations between internal and external locus of control and achievement motives, notably hope for success and fear of failure among women in the five aforementioned Arab countries.

Based on the above, we hypothesized that self-esteem would statistically mediate the associations between internal and external locus of control and achievement motives (hope for success and fear of failure) among women from five Arab countries.

## Methods

### Study design

This study employed a multinational cross-sectional design with an anonymous online survey conducted between July 2024 and March 2025 in five Arab countries: Lebanon, Oman, Egypt, Palestine and Morocco. Native Arabic-speaking women aged 18 years or older were invited to voluntarily participate in an online survey administered via Google Forms, with an average completion time of approximately 20 min. Prior to initiating the survey, participants were presented with an informed consent page clearly outlining the study’s aims, assurances of anonymity, and their right to withdraw at any time without consequence. Informed consent of volunteers was then obtained by choosing “yes” to the following question: “Do you agree to participate in this study?” thus taking them further into the survey. Data collection followed a convenience-based online approach and participation was voluntary with no compensation provided.

### Participants

Participants were recruited from Lebanon, Egypt, Oman, Palestine, and Morocco. These five countries were selected to represent geographic and sociopolitical diversity within the Arab region, while sharing Arabic language, cultural heritage and broadly collectivist social structures. An online survey was circulated by a snowball sampling technique, whereby the survey link was initially shared through personal and social networks. Respondents were encouraged to forward it to other eligible women. Eligibility criteria for this study were: Native Arabic speaking women aged 18 years or older and concurrently residing in one of the 5 included countries. A total of 1994 individuals accessed the survey. Twenty-four declined participation by not providing informed consent, and seven respondents were excluded because they were younger than 18 years of age. The final analytical sample consisted of 1963 eligible participants. To limit duplicate entries, the survey was restricted to one response per account.

### Sample size calculation

We used Fritz and MacKinnon’s recommendations (Fritz and Mackinnon, 2007) to calculate the minimal sample size. The minimum sample size was estimated at 413, considering a small effect size (*f* = 0.14), *L* = 7.85 for an alpha error of 0.05 and a statistical power of 80%, and 13 variables to be entered in the mediation models.

### Measures

Participants submitted a response to an anonymous online questionnaire in Arabic between July 2024 and March 2025. The questionnaire included two sections: sociodemographic information and standardized scales assessing locus of control, self-esteem, and achievement motives, described below. All measures were administered in Arabic using validated or psychometrically evaluated Arabic versions when available, as described below.

Sociodemographic characteristics and background information were provided by the participants including age, country, education level, work status, religion, and household crowding index. Household Crowding Index (HCI) was calculated by dividing the number of household residents by the number of rooms used for sleeping (persons-per-bedroom), with higher values indicating greater household crowding (Karam et al., 2024).

#### Single-item self-esteem scale (SISES)

Self-esteem was assessed using the Single-Item Self-Esteem Scale (SISES) which asks participants to rate their overall self-esteem with a single statement “I have high self-esteem” on a 5-point Likert scale ranging from 1 (not very true of me) to 5 (very true of me). Scores were recorded as the single item value (1–5), with higher scores indicating higher self-esteem. The Arabic version of the item has been used and psychometrically supported in Arabic-speaking samples (Fekih-Romdhane et al., 2023a).

#### Internal-external locus of control short scale-4 (IE-4)

Locus of control was evaluated using the 4-item Internal-External Locus of Control Short Scale (IE-4). The IE-4 is composed of two distinct subscales: internal locus of control and external locus of control, with two items on each subscale. The Arabic version has been validated in Arabic-speaking samples (Fekih-Romdhane et al., 2023b). Participants responded using a 5-point Likert scale ranging from strongly disagree to strongly agree. The internal locus of control items reflects the belief that outcomes depend on one’s own actions and efforts, whereas the external locus of control items reflect the belief that outcomes are shaped by factors outside one’s control, such as luck, fate or external circumstances. Internal and external locus-of-control scores were calculated separately by averaging the two items of each subscale, with higher scores indicating stronger endorsement of the corresponding orientation. In the present sample, internal consistency was low for both subscales: For the internal locus of control subscale, the Spearman-Brown Correlation Coefficient was 0.35 and the inter-item correlation between IE1 and IE2 was r = 0.211. For the external locus of control subscale, the Spearman-Brown Correlation Coefficient was 0.46 and the inter-item correlation between IE3 and IE4 was *r* = 0.300. Although low alpha values may occur in very brief scales because Cronbach’s alpha is partly influenced by the number of items, these coefficients remain below conventional acceptability thresholds and indicate limited reliability in this sample (Tavakol and Dennick, 2011). Therefore, all findings involving locus of control should be interpreted cautiously.

#### The revised achievement motives scale

Achievement motives were assessed using the revised 10-item Achievement Motives Scale originally developed by Lang and Fries, which measures two components: hope for success and fear of failure, with five items in each subscale (Lang and Fries, 2006). The Arabic version of this scale has been psychometrically validated in an Omani sample (Alsoudi et al., 2023). Example items include “I like situations in which I can find out how capable I am” for hope for success and “I feel uneasy to do something if I am not sure of succeeding” for fear of failure. Although the original instrument used a four-point response format, the Arabic version administered in the present study used a five-point Likert scale ranging from 1 (strongly disagree) to 5 (strongly agree). Subscale scores were calculated separately by summing the five items corresponding to each dimension, yielding possible scores ranging from 5 to 25 for both hope for success and fear of failure subscales, with higher scores indicating stronger endorsement of the corresponding motive. In the present study, Cronbach’s α = 0.92 for hope for success and α = 0.88 for fear of failure.

#### Perceived parental son preference

Participants were asked whether either of their parents had preferred a boy during pregnancy, with response options “yes,” “no” and “I do not know.” This study-specific item was included as a culturally relevant covariate reflecting participants perceived parental son preference.

### Statistical analysis

SPSS version 27 was used to analyze our data, while measurement invariance analyses were conducted in RStudio using the lavaan and semTools packages. Cronbach’s α values were used to check the internal reliability of the scales. The use of the Spearman-Brown Correlation Coefficient was recommended to test the reliability of the instruments consisting of two items (Eisinga et al., 2013). Hence, in the present study, the Spearman-Brown Correlation Coefficient was used to calculate the split-half reliability of the instrument. Normality was checked using skewness and kurtosis thresholds. Normality was considered acceptable when skewness and kurtosis with absolute skewness value < 2 and absolute kurtosis value < 7 were considered acceptable (Curran et al., 1996). Comparison of means was done by employing the student t-test and ANOVA. The association between continuous variables was done by using Pearson correlation test. Correction for multiple testing was done by dividing 0.05 by the number of variables tested (=13), yielding a corrected *p* = 0.0038. These included country, marital status, having children, religion, education, place of residence, work status, perceived parental son preference, self-esteem, internal locus of control, external locus of control, age, and household crowding. PROCESS Macro version 4.2 Model 4 was used to conduct mediation analyses, with indirect effects estimated using 5,000 bootstrap samples and 95% bootstrap confidence intervals. Thirty participants had missing data for perceived parental son preference. Missing data were handled using listwise deletion, as implemented by the PROCESS macro. Consequently, participants with missing data on any variable included in a given mediation model were excluded from that analysis. Covariates were identified *a priori* based on theoretical considerations and previous literature. These included age, having children, education, household crowding index, country of residence to account for contextual differences across participating countries, and perceived parental son preference as a culturally relevant contextual factor. Because hope for success and fear of failure represent related but distinct motivational outcomes, the primary mediation models used outcome-specific adjustment sets. This approach was intended to avoid unnecessary adjustments for variables that showed no evidence of association with a given outcome while preserving consistency with the outcome-specific bivariate analyses. To evaluate the robustness of these findings, sensitivity analyses were additionally performed using a common prespecified covariate set that included all measured demographic variables, irrespective of their statistical significance in the bivariate analyses. Statistical significance was set at *p* < 0.05 in the mediation models.

## Results

In total, 1963 participants completed the questionnaire, with 27.7% Lebanese females and a mean age of 27.42 years. The full description of the sample is in Table 1. The results of the measurement invariance of the scale across countries can be found in Supplementary Table S1.

**Table 1:** Sociodemographic and other characteristics of participants (*N* = 1963).

Variables	*N* (%)
Country	
Lebanon	543 (27.7%)
Oman	393 (20.0%)
Egypt	527 (26.8%)
Palestine	282 (14.4%)
Morocco	218 (11.1%)
Marital status	
Single, divorced, widowed	1,336 (68.1%)
Married, in a relationship	627 (31.9%)
Having Kids	
No	1,468 (74.8%)
Yes	495 (25.2%)
Religion	
Islam	1,525 (77.7%)
Christian	386 (19.7%)
Others	52 (2.6%)
Education level	
School level	124 (6.3%)
University level	1839 (93.7%)
Place of living	
Urban	1,354 (69.0%)
Rural	609 (31.0%)
Work status	
Unemployed	1,295 (66.0%)
Employed	668 (34.0%)
Either of your parents prefer a boy during pregnancy	
No	841 (42.8%)
Yes	473 (24.1%)
I do not know	619 (31.5%)

Variables	Mean ± SD
Age (years)	27.42 ± 10.77
Household Crowding Index (HCI)	1.34 ± 0.75
Achievement motive (hope for success)	19.20 ± 4.96
Achievement motive (fear of failure)	15.86 ± 5.36
Self-esteem	2.74 ± 1.11
Internal locus of control	3.30 ± 0.92
External locus of control	2.41 ± 0.92

### Bivariate analysis

Country and perceived parental son preference were significantly associated with both achievement motives scores in the omnibus analyses. Descriptively, participants from Oman had the highest mean hope for success score, whereas participants from Morocco had the highest mean fear of failure score. Participants reporting no perceived parental gender preference had the highest mean hope for success score, whereas those reporting perceived parental gender preference for a boy had the highest mean fear of failure score. Bonferroni-adjusted pairwise comparisons showed that women in Palestine had significantly lower hope for success scores than women in Lebanon (*p* = 0.031), Oman (*p* < 0.001) and Egypt (*p* < 0.001). No other country comparisons were significant for hope for success. Regarding fear of failure, women in Morocco had significantly higher scores than women in Lebanon (*p* < 0.001), Oman (*p* = 0.017), and Palestine (*p* < 0.001). Women in Egypt also had higher fear of failure scores than those in Lebanon (*p* = 0.007) and Palestine (*p* < 0.001), while women in Oman had higher scores than those in Palestine (*p* = 0.043). Perceived parental son preference was also significantly associated with both achievement motives scores. Women reporting no parental gender preference had significantly higher hope for success scores than those who did not know their parents’ preference (*p* < 0.001). Women reporting that either parent preferred a boy had significantly higher fear of failure scores than women with no preference (*p* = 0.017) and those who did not know their parents’ preference (*p* < 0.001). No significant difference was found between the “No” and “I don’t know” groups for fear of failure (Table 2).

**Table 2:** Bivariate analysis of factors associated with achievement motive scores.

	Hope for success score					Fear of failure score				
	Mean ± SD	*t*/*F*	df/df1, df2	*P*	Effect size	Mean ± SD	*t*/*F*	df/df1, df2	*P*	Effect size
Country		7.11	4, 1958	**<0.001**	0.014		10.34	4, 1958	**<0.001**	0.021
Lebanon	19.03 ± 4.85					15.40 ± 5.60				
Oman	19.88 ± 4.57					15.79 ± 5.22				
Egypt	19.54 ± 4.77					16.50 ± 5.23				
Palestine	17.96 ± 6.03					14.60 ± 5.41				
Morocco	19.14 ± 4.56					17.20 ± 4.81				
Marital status		0.15	1961	0.884	0.007		0.17	1961	0.865	0.008
Single, divorced, widowed	19.21 ± 5.03					15.87 ± 5.36				
Married, in a relationship	19.17 ± 4.83					15.83 ± 5.38				
Having Kids		0.58	1961	0.564	0.030		3.61	1961	**<0.001**	0.188
No	19.23 ± 4.91					16.11 ± 5.28				
Yes	19.08 ± 5.13					15.11 ± 5.54				
Religion		0.66	2, 1960	0.519	0.001		3.16	2, 1960	0.043	0.003
Muslim	19.14 ± 5.06					15.91 ± 5.38				
Christian	19.36 ± 4.64					15.46 ± 5.30				
Others	19.75 ± 4.23					17.35 ± 5.11				
Education level		2.25	136.95	0.026	0.228		0.72	1961	0.474	0.066
School level	18.14 ± 5.44					15.52 ± 5.70				
University level	19.27 ± 4.92					15.88 ± 5.34				
Place of living		0.72	1102.71	0.470	0.036		0.21	1961	0.837	0.010
Urban	19.25 ± 4.85					15.84 ± 5.33				
Rural	19.07 ± 5.20					15.89 ± 5.44				
Work status		2.03	1961	0.043	0.097		2.33	1961	0.020	0.111
Unemployed	19.03 ± 5.16					16.06 ± 5.32				
Employed	19.51 ± 4.55					15.47 ± 5.43				
Either of your parents prefer a boy during pregnancy		9.21	2, 1930	**<0.001**	0.009		8.48	2, 1930	**<0.001**	0.009
No	19.74 ± 4.40					15.81 ± 5.14				
Yes	19.09 ± 4.99					16.66 ± 5.21				
I do not know	18.64 ± 5.48					15.32 ± 5.63				

Bold numbers indicate significant *p*-value (*p* < 0.004) after Bonferroni correction. The reported effect sizes correspond to Cohen’s *d* values for dichotomous variables and partial eta squared for variables with 3 or more categories.

In addition, higher levels of self-esteem and internal locus of control were significantly associated with more hope for success, whereas higher internal and external locus of control were significantly associated with more fear of failure. Older age was significantly associated with less fear of failure (Table 3).

**Table 3:** Pearson correlation matrix.

Variables	1	2	3	4	5	6	7
1. Achievement motive (hope for success)	1						
2. Achievement motive (fear of failure)	0.37***	1					
3. Self-esteem	0.38***	−0.02	1				
4. Internal locus of control	0.40***	0.13***	0.30***	1			
5. External locus of control	0.03	0.28***	−0.08***	0.07**	1		
6. Age	−0.01	−0.11***	−0.04	0.09***	−0.09***	1	
7. Household Crowding Index	−0.04	−0.01	−0.04	−0.13***	0.03	−0.27***	1

*** indicate significant *p*-value (*p* < 0.004) after Bonferroni correction.

### Analysis of mediation

Because the data were cross-sectional, the mediation results are interpreted as statistical associations within the specified models and do not establish temporal ordering or causality. All mediation analyses were conducted on the same analytic sample (*n* = 1,933) after listwise deletion of cases with missing data on variables included in the models.

The mediation analysis taking the fear of failure score as the dependent variable, was adjusted for the following covariates: country, having kids, perceived parental gender preference and age. Higher internal locus of control was significantly associated with higher self-esteem (*B* = 0.18, *p* < 0.001). In turn, higher self-esteem was significantly associated with lower fear of failure (*B* = −0.34, *p* = 0.003). The total association between internal locus of control and fear of failure was statistically significant (*B* = 0.37, *p* < 0.001). However, after accounting for self-esteem, the direct association between internal locus of control and fear of failure remained statistically significant (*B* = 0.43, *p* < 0.001). The indirect association between internal locus of control and fear of failure through self-esteem was statistically significant (indirect effect = −0.06, 95% bootstrap CI [−0.10, −0.02]) (Table 4, Model 1).

**Table 4:** Mediation analyses results.

Path	Unstandardized beta *B*	Standard error	Standardized beta *β*	*t*	*p*	95% CI
Model 1: Indirect effect of self-esteem in the association between internal locus of control and fear of failure						
Internal locus of control → Self-esteem (path a)	0.18	0.01	0.30	14.02	<0.001	0.16, 0.21
Self-esteem → Fear of failure (path b)	−0.34	0.11	−0.07	−2.94	0.003	−0.56, −0.11
Internal locus of control → Fear of failure (direct effect, path c’)	0.43	0.07	0.15	6.36	<0.001	0.30, 0.57
Internal locus of control → Fear of failure (total effect, path c)	0.37	0.07	0.13	5.73	<0.001	0.25, 0.50
Internal locus of control → Self-esteem → Fear of failure (indirect effect)	−0.06	0.02	−0.02	–	–	−0.10, −0.02
Model 2: Indirect effect of self-esteem in the association between external locus of control and fear of failure						
External locus of control → Self-esteem (path a)	−0.05	0.01	−0.08	−3.63	<0.001	−0.08, −0.02
Self-esteem → Fear of failure (path b)	−0.01	0.11	−0.003	−0.11	0.910	−0.22, 0.20
External locus of control → Fear of failure (direct effect, path c’)	0.76	0.06	0.26	11.91	<0.001	0.63, 0.88
External locus of control → Fear of failure (total effect, path c)	0.76	0.06	0.26	11.97	<0.001	0.63, 0.88
External locus of control → Self-esteem → Fear of failure (indirect effect)	0.001	0.01	0.001	–	–	−0.01, 0.01
Model 3: Indirect effect of self-esteem in the association between internal locus of control and hope for success						
Internal locus of control → Self-esteem (path a)	0.18	0.01	0.30	13.85	<0.001	0.15, 0.20
Self-esteem → Hope for success (path b)	1.23	0.09	0.27	13.01	<0.001	1.04, 1.41
Internal locus of control → Hope for success (direct effect, path c’)	0.84	0.06	0.31	14.93	<0.001	0.73, 0.96
Internal locus of control → Hope for success (total effect, path c)	1.06	0.06	0.40	18.92	<0.001	0.95, 1.17
Internal locus of control → Self-esteem → Hope for success (indirect effect)	0.22	0.03	0.08	–	–	0.17, 0.27
Model 4: Indirect effect of self-esteem in the association between external locus of control and hope for success						
External locus of control → Self-esteem (path a)	−0.05	0.01	−0.08	−3.46	<0.001	−0.07, −0.02
Self-esteem → Hope for success (path b)	1.67	0.10	0.37	17.55	<0.001	1.48, 1.86
External locus of control → Hope for success (direct effect, path c’)	0.16	0.06	0.06	2.86	0.004	0.05, 0.27
External locus of control → Hope for success (total effect, path c)	0.08	0.06	0.03	1.39	0.163	−0.04, 0.20
External locus of control → Self-esteem → Hope for success (indirect effect)	−0.08	0.02	−0.03	–	–	−0.13, −0.03

The indirect effect through self-esteem was not statistically significant for the association between external locus of control and fear of failure (indirect effect: *B* = 0.001; 95% Boot CI −0.01; 0.01) (Table 4, Model 2).

The mediation analysis taking the hope for success score as the dependent variable, was adjusted for the following covariates: country and perceived parental gender preference. Higher internal locus of control was significantly associated with higher self-esteem (*B* = 0.18, *p* < 0.001). In turn, higher self-esteem was significantly associated with higher hope for success (*B* = 1.23, *p* < 0.001). The total association between internal locus of control and hope for success was statistically significant (*B* = 1.06, *p* < 0.001). However, after accounting for self-esteem, the direct association between internal locus of control and hope for success remained statistically significant (*B* = 0.84, *p* < 0.001). The indirect association between internal locus of control and hope for success through self-esteem was statistically significant (indirect effect = 0.22, 95% bootstrap CI [0.17, 0.27]) (Table 4, Model 3).

Higher external locus of control was significantly associated with lower self-esteem (Unstandardized Beta = −0.05, *p* < 0.001). In turn, higher self-esteem was significantly associated with higher hope for success (*B* = 1.67, *p* < 0.001). The total association between external locus of control and hope for success was not statistically significant (*B* = 0.08, *p* = 0.163). However, after accounting for self-esteem, the direct association between external locus of control and hope for success was statistically significant (*B* = 0.16, *p* = 0.004). The indirect association between external locus of control and hope for success through self-esteem was statistically significant (indirect effect = −0.08, 95% bootstrap CI [−0.13, −0.03]) (Table 4, Model 4).

The mediation analysis taking the fear of failure score as the dependent variable, was adjusted for the following covariates: country was entered as a categorical covariate using indicator variables, with Lebanon as the reference category, having kids (0 = no; 1 = Yes), Perceived parental son preference was entered as a categorical covariate using two indicator variables (“Yes” vs. “No” and “Do not know” vs. “No”), with “No” serving as the reference category.

The mediation analysis taking the hope for success score as the dependent variable, was adjusted for the following covariates: country was entered as a categorical covariate using indicator variables, with Lebanon as the reference category, perceived parental son preference was entered as a categorical covariate using two indicator variables (“Yes” vs. “No” and “Do not know” vs. “No”), with “No” serving as the reference category.

### Sensitivity analysis

As a sensitivity analysis, sociodemographic variables considered theoretically relevant to the modeled associations were entered simultaneously as covariates, irrespective of their statistical significance in the bivariate analyses. These variables included country, marital status, having children, religion, place of residence, employment status, parental gender preference, age and household crowding index.

For fear of failure, the indirect association of internal locus of control through self-esteem remained statistically significant (*B* = −0.06; BootSE = 0.02, 95% bootstrap CI [−0.11, −0.02]). In contrast, the association of external locus of control through self-esteem remained non-significant (*B* = 0.001, BootSE = 0.01, 95% bootstrap CI [−0.01, 0.01]).

For hope for success, the indirect association of internal locus of control through self-esteem remained statistically significant (*B* = 0.22; BootSE = 0.03, 95% bootstrap CI [0.17, 0.28]). The association of external locus of control through self-esteem also remained statistically significant (*B* = −0.08, BootSE = 0.03, 95% bootstrap CI [−0.13, −0.03]). Thus, the direction, magnitude, and statistical significance of the indirect associations were unchanged after broader covariate adjustment.

## Discussion

This study examined whether self-esteem statistically mediates the association between locus of control and achievement motives among women drawn from five Arab countries, situating this inquiry within a cultural context characterized by collectivist values and gendered social structures that may be relevant to how control beliefs, self-evaluation, and achievement motivation are associated. Overall, statistically significant indirect associations through self-esteem were observed, particularly hope for success, while also showing that the pattern of this mediation differs depending on the locus dimension and the motivational outcome considered. Beyond the mediation patterns, bivariate analyses revealed associations between perceived parental son preference and achievement motivation, providing preliminary evidence of an association between perceived parental son preference and women’s motivational profiles. These findings are discussed in light of the cultural and theoretical framework outlined above.

The effect sizes should be interpreted cautiously, as several associations were small in magnitude and the cross-sectional design does not allow conclusions about causal or temporal relationships.

### Locus of control and self-esteem

In the adjusted mediation models, locus of control was associated with self-esteem, but in opposite directions depending on the locus dimension. However, these findings should be interpreted cautiously because the IE-4 subscales used in this study included only two items each and showed low internal consistency (Tavakol and Dennick, 2011). With this limitation in mind, higher internal locus of control was associated with higher self-esteem, whereas higher external locus of control was associated with lower self-esteem. This pattern is consistent with the idea that internal control beliefs may be associated with a more positive self-view, potentially because individuals who endorse such beliefs perceive themselves as capable of influencing outcomes through their own actions and efforts. Conversely, a stronger external orientation may be associated with lower self-esteem because outcomes may feel less connected to one’s own choices, which may be associated with a weaker relationship between everyday successes and perceived self-worth (Hosseini et al., 2016). Nevertheless, because of the limited reliability of the locus of control subscales, the magnitude and stability of these associations should not be overinterpreted.

Interestingly, this pattern emerged in a sample drawn from Arab countries where previous theoretical work has suggested that beliefs related to religiosity, family obligations, and interpersonal responsibility may be associated with perceptions of personal control (Dwairy et al., 2006; Zakiy et al., 2024). Although these cultural influences were not directly assessed in the present study, the observed association between external locus of control and lower self-esteem is consistent with the possibility that perceived control retains psychological importance even within cultural contexts where external attributions may be more socially normative.

### Self-esteem and achievement motives

Self-esteem was also associated with achievement motives. Higher self-esteem was associated with higher hope for success, and it was also associated with lower fear of failure. This pattern is theoretically consistent with the possibility that a more positive global self-evaluation is associated with greater confidence and persistence in achievement situations and with lower perceived threat in such situations (Li et al., 2022). By contrast, lower self-esteem may be associated with evaluative situations being perceived as riskier and more self-relevant, as well as with greater sensitivity to mistakes and fear-based, avoidance-leaning reactions (Li et al., 2022). One possible interpretation is that, in Arab cultural contexts where previous research has suggested that self-esteem is more relationally grounded (Dwairy et al., 2006; Markus and Kitayama, 1991), social validation and family approval may be associated with motivational confidence (Du et al., 2017).

### Direct associations of locus of control and achievement motives

Beyond self-esteem, the locus of control indicators retained direct associations with achievement motives in some models. These findings, however, should be read cautiously because the internal and external locus of control subscales were very brief and showed low internal consistency (Tavakol and Dennick, 2011). Internal locus of control was associated with higher hope for success, suggesting that women who more strongly endorsed personal control over outcomes also tended to report stronger approach-oriented achievement motivation (Judge and Bono, 2001). At the same time, internal locus of control was positively associated with fear of failure. One possible interpretation is that a stronger sense of personal responsibility may be associated with greater perceived pressure in evaluative situations: when outcomes are viewed as “on me,” failure may be perceived as more personally consequential (Sagar and Stoeber, 2009). However, given the measurement limitation, this interpretation should be considered tentative and requires replication using more comprehensive locus-of-control measures.

The findings for external locus of control also require caution: Although external locus of control was indirectly associated with lower hope for success through lower self-esteem, the direct association with hope for success was positive after accounting for self-esteem. This mixed pattern may be consistent with a possible role of external expectations, family approval or social incentives in achievement striving among Arab women (Borham et al., 2023). In other words, some women may remain motivated to succeed because achievement is socially valued or expected, even when their own self-evaluation is weaker. Still, because the external locus of control subscale consisted of only two items and showed low internal consistency, this explanation remains provisional.

### Mediating role of self-esteem

Overall, the indirect associations indicate that self-esteem may statistically account for part of the associations between locus of control and achievement motives (Skinner, 1996).

For hope for success, statistically significant indirect associations through self-esteem were observed for both internal and external locus of control. Specifically, the indirect effect was significant for both internal and external locus of control. In practical terms, this pattern indicates that the association between stronger internal orientation and higher hope for success included an indirect association through self-esteem (Judge and Bono, 2001). Notably, the external locus model also suggested that the direct and indirect effects operated in opposite directions; this means that externally oriented individuals may report lower self-esteem that is associated with lower hope, while external pressures or incentives may also be associated with achievement striving through an association independent of the indirect association involving self-esteem (Graham et al., 2022).

For fear of failure, self-esteem showed a different pattern. It statistically accounted for part of the association with internal locus of control, indicating a pattern in which internal control was associated with higher self-esteem, while higher self-esteem was associated with lower fear of failure, even though internal locus of control still retained a direct positive association with fear of failure. This interpretation should also be considered tentative because the observed effects were modest and the study design does not establish directionality.

In contrast, self-esteem did not statistically account for the association between external locus of control and fear of failure, which suggests that other factors such as uncertainty, anxiety or concerns about social evaluation may also be associated with fear-based motivation among individuals with stronger external control beliefs (Graham et al., 2022).

Therefore, these statistically significant indirect associations should be interpreted as modest preliminary patterns rather than strong practical effects.

### Country level variation

Variation in achievement motivation scores was found between countries. Significant differences emerged with Moroccan women showing the highest fear of failure scores and Palestinian women the lowest hope for success scores. While countries were included as covariate rather than a primary variable, these differences may be associated with differences in the sociopolitical, economic and cultural conditions of each context, including differing levels of political instability, education opportunity and prevailing gender norms. Future research would benefit from examining country-level moderators explicitly (Judge and Bono, 2001).

### Practical implications

Given the cross-sectional design, the present findings should not be interpreted as evidence that modifying self-esteem or control beliefs would directly change achievement motivation. Nevertheless, the observed associations may help identify areas for future prevention and support efforts among Arab women. In particular, the association between self-esteem and higher hope for success, together with lower fear of failure, suggests that self-evaluation may be a relevant target for future longitudinal and intervention-based research (Sowislo and Orth, 2013). Similarly, the mixed pattern observed for locus of control suggests that perceived personal responsibility may be linked to both approach-oriented motivation and performance-related pressure, although this interpretation remains tentative, especially given the low reliability of the brief locus-of-control subscales (Judge and Bono, 2001).

These findings may be useful for generating hypotheses for culturally responsive support programs that consider both individual self-evaluation and the relational context in which achievement occurs (Heine et al., 1999). However, the present study did not test counseling strategies; therefore, any intervention-related recommendations should be considered preliminary. Longitudinal and experimental studies are needed to determine whether interventions targeting self-esteem, control beliefs or family-level support are associated with changes in achievement motivation or fear of failure among Arab women.

### Strengths and limitations

This multinational design contributes to the cross-cultural psychology literature by examining constructs like locus of control, self-esteem and achievement motivation that have been predominantly studied in Western, individualist samples, extending the examination of these associations to a multinational sample of women from five Arab countries.

However, several limitations should be considered. First, due to the cross-sectional design of this study, causal or temporal conclusions cannot be drawn. Consequently, it remains unclear whether control beliefs precede self-esteem and motivation, or whether motivational tendencies precede control beliefs and self-evaluation or whether these associations are bidirectional over time. Second, all variables were assessed via self-report measures which may introduce shared method variance, social desirability effects and reporting biases. It was also not possible to objectively verify participants’ responses. Third, the online convenience approach with snowball sampling used to recruit participants may have led to a sample reflecting women who are connected to online and social networks and willing to participate in surveys, which can increase selection bias and limit generalizability to women with more restricted internet access or lower social media engagement (Chen et al., 2021). In addition, the sample was highly educated, with 93.7% of participants reporting university-level education. This differs from the educational distribution of the general female population in the five included countries and should be considered when interpreting the findings. The restricted educational range may have reduced variability in achievement-related experiences, self-evaluation and perceived control, which could have attenuated some associations or produced estimates that are more reflective of educated, socially connected women than of Arab women more broadly (Lakes, 2013). At the same time, higher education may be associated with greater exposure to achievement settings and stronger awareness of personal agency, so the observed relationships between locus of control, self-esteem and achievement motives may not operate in the same way among women with lower educational attainment. Therefore, the findings should not be generalized to the general female population of these countries without caution. Future studies should use more diverse sampling strategies and intentionally recruit women across different educational levels, socioeconomic backgrounds and rural/urban settings.

In addition, and despite including covariates, residual confounding factors may still be present due to unassessed factors such as mental health symptoms or broader personality traits which may also be associated with self-esteem and achievement motives. Moreover, all variables were self-reported and collected simultaneously using a single survey platform, which may have introduced common method variance. However, the presence of statistically significant associations for some tested indirect models but not others suggests that common method variance alone is unlikely to account for all of the observed findings, as self-esteem significantly mediated some relationships, whereas no mediation was observed for the association between external locus of control and fear of failure. If common method variance were the primary explanation, stronger and more uniform associations across all tested models would generally be expected. Another important limitation concerns the measurement of locus of control. The IE-4 is a very brief instrument composed of two separate subscales, internal and external locus of control, with only two items per subscale. In the present sample, both subscales showed low internal consistency. For internal locus of control, Cronbach’s α was 0.34 and the inter-item correlation between IE1 and IE2 was *r* = 0.211. For external locus of control, Cronbach’s α was 0.46 and the inter-item correlation between IE3 and IE4 was *r* = 0.300. In addition to indicating limited reliability, the low inter-item correlations suggest that the two items within each IE-4 subscale may not adequately reflect the same underlying dimension, raising concerns about the extent to which these brief subscales capture the intended locus-of-control constructs in the present sample. Although low Cronbach’s alpha values may occur in very short scales because alpha is partly influenced by the number of items, these coefficients remain below conventional acceptability thresholds and indicate limited reliability in this sample (Tavakol and Dennick, 2011). Furthermore, measurement invariance of the IE-4 was not supported across the five countries. Specifically, the internal locus of control showed weak measurement properties in the Moroccan sample, preventing the establishment of configural invariance across all five countries. Additional diagnostic analyses excluding Morocco also failed to support full measurement invariance across Lebanon, Oman, Egypt, and Palestine. Consequently, observed cross-country differences in locus-of-control scores may partly reflect differences in how the construct is measured or interpreted across countries rather than true differences in the underlying constructs. Cross-country comparisons involving locus of control should therefore be interpreted cautiously. This is important because locus of control was the primary independent variable in all mediation models. Limited reliability and the possibility that the brief subscales did not fully capture the intended constructs may introduce measurement error that attenuates associations, reduces statistical power and makes estimated coefficients less stable. Therefore, the direct and indirect statistical associations involving locus of control may be attenuated or imprecisely estimated. For this reason, all findings involving locus of control should be interpreted cautiously and considered preliminary. Future studies should replicate the present mediation models using longer and psychometrically stronger measures of internal and external locus of control^.^

Future research would benefit from longitudinal studies to examine the temporal ordering of locus of control, self-esteem and achievement motives and determine whether the patterns observed remain stable over time. It would also be useful to incorporate multi-method assessment (behavioral indicators of persistence, performance-based tasks or qualitative interviews) to complement self-report and reduce shared method bias. Another limitation is the use of a single-item measure of self-esteem. Although the SISE has demonstrated acceptable reliability and validity and has been widely used to assess global self-esteem, it may not capture the multidimensional nature of the construct. In particular, because the present study is grounded in a theoretical framework emphasizing the relevance of family dynamics, cultural norms and gender-related experiences, a single global item may not fully reflect relational and culturally embedded aspects of self-esteem. Consequently, the observed mediation effects should be interpreted with caution. Given that self-esteem did not statistically account for the external locus–fear of failure association, future studies could also test alternative variables such as uncertainty, anxiety, perceived uncontrollability and social-evaluation concerns, which may also be associated with fear-based motivational patterns in externally oriented individuals. The multinational design represents both strength and limitations. Although the countries included have several broad cultural characteristics that justify their examination within a common regional framework, they also differ substantially in their sociopolitical environments, economic conditions, and gender norms. Consequently, the present findings should not be interpreted as implying cultural uniformity across Arab societies. Rather, they suggest that the observed associations emerged across a culturally diverse regional sample, while future research should explicitly investigate country-specific differences and potential cultural moderators. Moreover, although the overall sample largely exceeded the required minimum, the country-specific sample sizes were not all sufficient for adequately powered mediation analyses. In particular, three of the five participating countries did not reach the estimated minimum sample size required for the mediation model. Future studies should recruit sufficiently large samples within each country to examine whether the observed mediation patterns are consistent across different Arab populations. Finally, although our interpretation draws on theoretical perspectives related to collectivism, religiosity, family expectations, and relational self-esteem, these constructs were not directly assessed. Accordingly, these interpretations should be viewed as theoretically informed contextual interpretations rather than empirically tested mechanisms.

## Conclusion

This multinational cross-sectional study investigated how locus of control is associated with achievement motives and whether the association between locus of control and achievement motives includes a statistically significant indirect association through self-esteem among Arab women across five countries. Overall, the results demonstrated statistically significant indirect associations involving self-esteem, particularly for hope for success. Those findings support a nuanced view: both self-evaluative processes and pressure-related dynamics may be associated with achievement motivation among Arab women, with the statistical indirect associations involving self-esteem being particularly evident for hope for success. Longitudinal studies are needed to clarify temporal ordering and to examine additional variables that may also be associated with fear-based motivation among individuals with stronger external control beliefs.

## Data Availability

The raw data supporting the conclusions of this article will be made available by the authors, without undue reservation.
