# Supplementary material for: The mediating role of self-esteem in the association between locus of control and fear of failure and hope for success among women in five Arab countries
Source: Front Psychol. 2026 Oct 21;17:1890383. doi: 10.3389/fpsyg.2026.1890383 (PMC13730950; doi:10.3389/fpsyg.2026.1890383)
Supplement: Supplementary file 1 [file Table_1.docx]

Measurement invariance across countries was examined using multigroup confirmatory factor analysis (CFA). Given the ordinal nature of the items, models were estimated using the weighted least squares mean- and variance-adjusted (WLSMV) estimator with theta parameterization. For the Achievement Motives Scale, measurement invariance across Lebanon, Oman, Egypt, Palestine, and Morocco was evaluated sequentially at the configural, metric, scalar, and strict levels. For the ordered categorical indicators, threshold invariance was tested as an intermediate step before imposing equality constraints on factor loadings, following the identification approach proposed by Wu and Estabrook (2016). Measurement invariance was evaluated primarily based on changes in approximate fit indices between successive models. Changes of ≤ .010 in the comparative fit index (CFI) and ≤ .015 in the root mean square error of approximation (RMSEA) were considered indicative of invariance, together with changes in the standardized root mean square residual (SRMR) of ≤ .030 for metric invariance and ≤ .010 for scalar and strict.

**Results**

Measurement invariance of the Achievement Motives Scale and the Internal-External Locus of Control Short Scale-4 (IE-4) (Supplementary Table S1). For the Achievement Motives Scale, the configural model showed CFI = .984, TLI = .978, RMSEA = .109, and SRMR = .058. Although the RMSEA of the configural model was elevated, progressively imposing equality constraints resulted in no meaningful deterioration in approximate fit indices. Metric, scalar, and strict invariance were therefore supported across Lebanon, Oman, Egypt, Palestine, and Morocco. In contrast, measurement invariance of the IE-4 was not supported across the five countries, χ²(15) = 64.69, CFI = .895, TLI = .790, RMSEA = .092, 90% CI [.070, .116], SRMR = .053. In particular, the Internal Locus of Control factor was not adequately supported in Morocco, where the standardized loadings of IE1 and IE2 were λ = .106 (p = .753). therefore, metric, scalar, and strict invariance of the IE-4 were not evaluated across the five countries. Additional diagnostics analyses restricted to Lebanon, Oman, Egypt, and Palestine also did not support full threshold invariance ($\Delta$CFI = -.704, $\Delta$RMSEA = .029, $\Delta$SRMR = .006).

Measurement invariance of the Internal-External Locus of Control Short Scale-4 (IE-4) was examined separately because each of its two dimensions is represented by only two indicators^1,2^. To identify these two-indicator factors, the unstandardized factor loadings of the two indicators within each factor were constrained to equality. Country-specific models were examined before cross-country equality constraints were imposed. When invariance across all five countries was not supported, additional diagnostic analyses were conducted to identify the sources of non-invariance.

**Supplementary Table S1. Measurement invariance of the Achievement Motives Scale and the Internal-External Locus of Control Short Scale-4 (IE-4) across countries.**

| **Scale** | **Invariance model** | **χ²** | **df** | **CFI** | **TLI** | **RMSEA (90% CI)** | **SRMR** | $\boldsymbol{\Delta}$**CFI** | $\boldsymbol{\Delta}$**RMSEA** | $\boldsymbol{\Delta}$**SRMR** |
| --- | --- | --- | --- | --- | --- | --- | --- | --- | --- | --- |
| **Achievement Motives Scale** | Configural | 904.59 | 170 | .984 | .978 | .109 [.102, .116] | .058 | - | - | - |
|  | Metric | 1078.15 | 282 | .982 | .986 | .088 [.083, .094] | .059 | .001 | .004 | .001 |
|  | Scalar | 1104.95 | 314 | .982 | .987 | .083 [.078, .089] | .059 | <.001 | .005 | <.001 |
|  | Strict | 1321.41 | 354 | .978 | .986 | .087 [ .082, .092] | .064 | .004 | .004 | .005 |
| **IE-4** | **Configural** | **64.69** | **15** | **.895** | **.790** | **.092 [0.70, .116]** | **.053** | **-** | **-** | **-** |

**Note.** Configural invariance of the IE-4 was not supported across the five countries. In Morocco, the standardized loadings of IE1 and IE2 om the Internal Locus of Control factor were λ = .106 (p = .753). Therefore, metric, scalar, and strict invariance were not evaluated across the five countries.

**References**

1. Chen FF. Sensitivity of goodness of fit indexes to lack of measurement invariance. structural equation modeling: A multidisciplinary journal, 14(3), 464–504. <https://doi.org/10.1080/10705510701301834>.

2. Cheung, & Rensvold. Evaluating goodness-of-fit indexes for testing measurement invariance. structural equation modeling. *A Multidisciplinary Journal*. :9(2), 233–255. <https://doi.org/10.1207/S15328007SEM0902_5>.
